# Prolonged response time helps eliminate residual errors in visuomotor adaptation

**DOI:** 10.3758/s13423-020-01865-x

**Published:** 2021-01-22

**Authors:** Lisa Langsdorf, Jana Maresch, Mathias Hegele, Samuel D. McDougle, Raphael Schween

**Affiliations:** 1 NemoLab - Neuromotor Behavior Laboratory, Department of Sport Science, Justus Liebig University, Giessen, Germany; 2 Center for Mind, Brain and Behavior (CMBB), Universities of Marburg and Giessen, Giessen, Germany; 3 Department of Psychology and Sport Science, Justus Liebig Unversity, Giessen, Germany; 4 Department of Brain and Cognitive Sciences, Ben-Gurion University of the Negev, Beersheva, Israel; 5 Department of Psychology, Yale University, New Haven, CT USA; 6 Department of Psychology, Philipps-University, Marburg, Germany

**Keywords:** Sensorimotor adaptation, Response time, Motor planning, Asymptote, Explicit strategies

## Abstract

One persistent curiosity in visuomotor adaptation tasks is that participants often do not reach maximal performance. This incomplete asymptote has been explained as a consequence of obligatory computations within the implicit adaptation system, such as an equilibrium between learning and forgetting. A body of recent work has shown that in standard adaptation tasks, cognitive strategies operate alongside implicit learning. We reasoned that incomplete learning in adaptation tasks may primarily reflect a speed-accuracy tradeoff on time-consuming motor planning. Across three experiments, we find evidence supporting this hypothesis, showing that hastened motor planning may primarily lead to under-compensation. When an obligatory waiting period was administered before movement start, participants were able to fully counteract imposed perturbations (Experiment 1). Inserting the same delay between trials – rather than during movement planning – did not induce full compensation, suggesting that the motor planning interval influences the learning asymptote (Experiment 2). In the last experiment (Experiment 3), we asked participants to continuously report their movement intent. We show that emphasizing explicit re-aiming strategies (and concomitantly increasing planning time) also lead to complete asymptotic learning. Findings from all experiments support the hypothesis that incomplete adaptation is, in part, the result of an intrinsic speed-accuracy tradeoff, perhaps related to cognitive strategies that require parametric attentional reorienting from the visual target to the goal.

## Introduction

One of the persistent curiosities in studying the human mind is the idea of canonical computations, that is that the brain applies similar computations to perform a wide range of different tasks. Most examples for such canonical computations (e.g., Carandini & Heeger, 2011; DiCarlo & Johnson, 2000; Miller, 2016; Movshon et al., 1978; Pack & Bensmaia, 2015; Ringach & Malone, 2007) have been identified in the fields of neuroscience and artificial intelligence but have largely eluded scientists in psychology.

One example of a reliable law in psychology is the speed-accuracy tradeoff, the inverse relation between the accuracy of an action and the time taken to produce it (for a review, see Heitz, 2014). The speed-accuracy tradeoff has been shown to shape behavior across domains from motor control (Fitts, 1954; Plamondon & Alimi, 1997) and perception (Grosjean et al., 2007) to memory (Hacker, 1980) and mental imagery (Cerritelli et al., 2000), as well as across species from insects (e.g., Ings & Chittka, 2008) and rodents (e.g., Rinberg et al., 2006) to monkeys (Heitz & Schall, 2012) and humans (Wickelgren, 1977).

Another example is the law of practice, according to which performance improvements are generally larger early during practice before they become systematically smaller as practice progresses giving rise to a negatively accelerated relationship between performance and the number of practice trials (Crossman, 1959, Chen et al., 2005). Regardless of its actual parameters, all versions of the law of practice postulate that performance improvements asymptote at some point. While it is almost impossible to determine the absolute maximum level of performance for complex skills such as swimming, in experimental paradigms like visuomotor transformation tasks (e.g., force field adaptation or rotations of visual feedback), individual performance improvements are evaluated relative to an absolute maximum. That is, there is a quantifiable level of complete adaptation to the transformation (Shadmehr, Brashers-Krug, & Mussa-Ivaldi, 1994).

Interestingly, one common observation in this context is that of an incomplete asymptote: If individuals are required to make reaching movements while compensating for a visuomotor rotation, their performance curve tends to asymptote below full compensation (Holland et al., 2018; Huberdeau et al., 2015; Haith et al., 2015; van der Kooij et al., 2016), leaving a residual performance error significantly different from zero (Hinder et al., 2010; Shmuelof et al., 2012; Spang et al., 2017; van der Kooij et al., 2015; Vaswani et al., 2015).

One approach to explain this is to leverage state-space models of adaptation, which are incremental Markovian learning algorithms that balance both learning and forgetting during adaptation (Smith et al., 2006). When fit to human learning data, most parameter values can produce a steady-state equilibrium at an arbitrary asymptote. Consequently, these models provide a natural description of the commonly observed undershoot, via an assumption that some amount of forgetting (i.e., reversion to baseline) is inevitable on each trial of the task. This interpretation suggests that incomplete compensation during motor learning is simply a built-in feature of the underlying learning mechanism.

However, Vaswani et al. (2015) demonstrated that humans, in principle, possess the capacity to overcome this incomplete asymptote. In their study, the cursor controlled by the participant moved in a fixed trajectory toward the target or to a nearby location with participants only controlling the amplitude. If the trajectory of the cursor had no variability, individuals appeared to adopt a new learning strategy that allowed them to fully counteract a novel visuomotor transformation. The authors proposed that this exploratory learning mechanism is typically suppressed by error-based learning. The putatively suppressed process only contributes to performance when error-based learning is disengaged, which in their study was caused by a persistent residual error in combination with a contextual change (i.e., the introduction of a lack of natural movement variability).

In the present study, we examined an alternative account of how humans might overcome incomplete asymptotic performance, where the level of performance achieved at later stages of visuomotor adaptation primarily reflects an intrinsic speed-accuracy tradeoff driven by time-consuming movement planning.

In line with this, research in perceptual decision-making has established that choice reaction time reflects a tradeoff between waiting for more information and acting early in order to speed up the accumulation of (uncertain) rewards on future trials (Churchland et al., 2008; Cisek et al., 2009; Thura et al., 2012; Thura & Cisek, 2017). While visuomotor adaptation tasks traditionally are not studied in the framework of decision-making, recent research has highlighted an important role for volitional decision-making strategies in adaptation tasks (i.e., the explicit re-aiming of movements to counteract perturbations; Bond & Taylor, 2015; Heuer & Hegele, 2009; Heuer & Hegele, 2015; McDougle et al., 2015; Schween & Hegele, 2017; Taylor et al., 2014). Further evidence suggests that in the context of adaptation to a novel visuomotor rotation, such strategies may take the form of mentally rotating the aiming direction of the reaching movement (McDougle & Taylor, 2019), which has been known to require long preparation times (Fernandez-Ruiz et al., 2011; Haith et al., 2015; McDougle & Taylor, 2019). Thus, an incomplete learning asymptote could arise from hurried movement initiation leading to prematurely terminating mental rotation of an abstract aiming trajectory during movement planning (Leow et al., 2017).

We tested our hypothesis over three behavioral experiments where we artificially extended planning time. We predicted that this simple manipulation would alleviate incomplete asymptotic learning (i.e., asymptotic reaching angles that undershoot the ideal angle). In Experiment 1, we introduced a mandatory waiting period between target presentation and movement onset. In Experiment 2, we sought to exclude effects of the total experiment duration by emphasizing the role of within-trial movement planning time versus between-trial consolidation. Finally, in Experiment 3, we used an aiming report method (Taylor et al., 2014) to promote the application of explicit motor learning strategies before movement execution and elucidated their influence on the learning asymptote.

## General methods

### Participants

A total of 90 neurologically healthy, right-handed students (Experiment 1: N = 36, Experiment 2: N = 36, Experiment 3: N = 18) from the Justus Liebig University Giessen participated in this study. They were recruited as participants and received monetary compensation or course credit for their participation. Written informed consent was obtained from all participants before testing. The experimental protocol was approved by the local ethics committee of the Department of Psychology and Sport Science.

### Apparatus

**Fig. 1:**
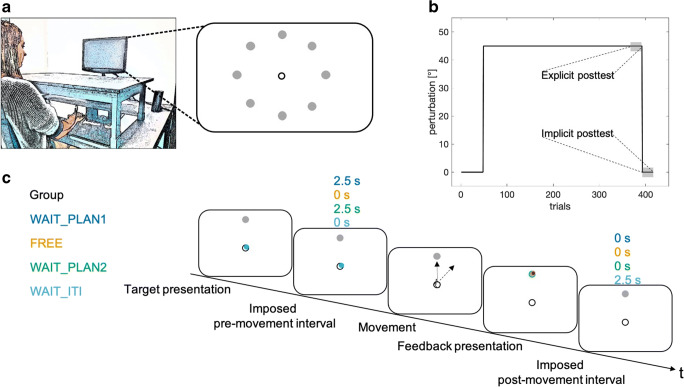
Schematic display of the experimental setup (**A**), overall protocol (**B**), and sequence of one trial (**C**). Each participant performed center-out reaching movements with a stylus on the tablet. Visual stimuli and the cursor were presented on a monitor. The visual cursor was displaced according to the protocol (**B**). During baseline, cursor and stylus position were veridical, during adaptation, the cursor was rotated 45°clockwise relative to the stylus position. Within-trial timing differed between groups (**C**). Group-dependent differences within one trial occurred during either the pre- or the post-movement interval. Whereas the FREE and WAIT_ITI groups had no specific task during the pre-movement interval, WAIT_PLAN1 and WAIT_PLAN2 groups were required to wait 2.5 s. During the post-movement interval, only the participants in the WAIT_ITI group were required to wait 2.5 s, whereas all other groups continued with the next trial immediately. The AIM group is not presented in this figure as their manipulation did not refer to any time constraints. Panel A is adapted from Schween, Taylor, and Hegele (2018) under CC-BY-4.0 license

Participants sat on a height-adjustable chair facing a 22-in. widescreen LCD monitor (Samsung 2233RZ; display size: 47.3 cm × 29.6 cm; resolution: 1,680 × 1,050 pixels; frame rate 120 Hz), which was placed at eye level 100 cm in front of them. Their right hand held a digitizing stylus, which they could move across a graphics tablet (Wacom Intuos 4XL). Their hand position recorded from the tip of the stylus was sampled at 130 Hz. Stimulus presentation and movement recording were controlled by a custom-built MATLAB script (R2017b), displayed above the table platform, thus preventing direct vision of the hand (left panel Fig. 1A).

### Task

Participants performed center-out reaching movements from a common start location to targets in different directions. They were instructed to move the cursor as quickly as possible from the start location in the direction of the displayed target and “shoot through it.” On the monitor, the start location was in the center of the screen, marked by the outline of a circle of 7 mm in diameter. On the table surface, the start location was 20–25 cm in front of the participant on the body midline. The screen target location, marked by a filled green circle of 4 mm in diameter, varied from trial to trial. Targets were placed on an invisible circle with a radius of 100 mm around the start location; target directions were 0°, 45°, 90°, 135°, 180°, 225°, 270°, and 315° (0° is from the start location to the right, 90° is forward, 270° is backward; right panel Figure 1A). On baseline and adaptation trials, visual feedback was given by a cursor (filled white circle, radius 2.5 mm).

### Design and procedure

The experiment consisted of three phases: baseline training, training with a 45° clockwise (CW) visuomotor rotation, and post-tests (Fig. 1B). Baseline training had veridical hand-cursor mapping and was organized into three blocks of eight trials each. Each block consisted of a random permutation of the eight target directions without any direction being repeated in successive trials. Training of the visuomotor rotation of 45° CW consisted of 40 blocks of eight trials each.

The post-test phase consisted of two types of trials: an explicit test (see below) comprising three blocks of eight trials each with each target location occurring once per block, and three blocks of eight after-effect test trials without visual feedback, with the instruction that the cursor rotation would be absent. In the explicit test trials (Hegele & Heuer, 2010; Heuer & Hegele, 2008), start and target locations were presented together with a white line, centered in the start location with its length corresponding to target distance. Initially, the line was presented at an angle of 180° CCW of the respective target’s direction. Participants instructed the experimenter to adjust the orientation of the line to match the direction of the movement they judged to be correct for the particular target presented.

Each single-movement trial started with the presentation of the start circle in the center of the screen, serving as the starting position for the subsequent reaching movement. In order to help guide participants’ movements back to the start, a white concentric circle appeared after feedback presentation, scaling its radius based on the cursor’s distance from the start circle. The cursor was displayed when it was within 3 mm of the start location. Once the start position was held for 300 ms, a tone (440 Hz, 500-ms duration) was presented, followed by a target appearing in one of the eight target positions and the start circle disappeared.

The cursor was visible until it exceeded a movement amplitude of 3 mm, after which it disappeared. When the participant’s hand crossed an invisible circle that contained the target, the cursor froze and reappeared in red, providing endpoint feedback for 1,250 ms. Movements that fell outside the range of instructed movement time (MT) criteria (MT < 100 ms or > 300 ms) were followed by an error message on the screen and the trial was aborted. Those trials were neither repeated nor used in subsequent analyses. If participants moved too soon in one of the waiting groups (before the appearance of the target or the go cue, see below), they were reminded to wait and the trial was repeated.

## Data analysis

The position of the stylus on the tablet surface was recorded and each trial was separately low-pass filtered (fourth-order Butterworth, 10 Hz) using Matlab’s *filtfilt* command and then numerically differentiated. Tangential velocity was calculated as the Euclidean distance of x- and y-velocity vectors. Behavior was analyzed in terms of two parameters: response time and endpoint error measured as final hand position. Response time was calculated as the interval between target presentation and movement onset, which was defined when tangential velocity exceeded 30 mm/s for at least five frames (38.5 ms). Endpoint error was calculated as the angular difference between the vector connecting the start circle and the target, and the vector connecting the start circle and the endpoint position. Endpoint errors were calculated for both training trials and the after-effect trials. The outcome variable of the explicit perceptual judgment test was calculated as the angular difference between the participant-specified line orientation on the screen and the vector connecting the start and target positions.

For each block of training trials and for the post-test, means were computed for each participant following screening for outliers. This screening ensured that single outlier movements were excluded before further analysis. Movements whose endpoint fell outside three standard deviations of the participants’ individual mean endpoint in that phase were considered outliers and removed. A total of 1.08% of all trials was detected and eliminated this way. To compare different levels of asymptote, the last five blocks of the training phase were median averaged and compared between groups using a two-sample Wilcoxon’s rank-sum test. To interpret the results, an effect size r and its 95% confidence interval were calculated. Statistical analyses were done in Matlab (R2017b) and R (version 3.5.1, http://www.R-project.org/). As a normal distribution was not always observed, all results are based on nonparametric tests.

## Experiment 1

According to the speed-accuracy tradeoff hypothesis, we expected prolonging response times to have a facilitating effect on adaptation. Experiment 1 investigated this hypothesis by manipulating participants’ response times in two groups and comparing their results. We predicted that the dependent variable (final hand position) would display less asymptotic error in a group in which response time was prolonged by the manipulation, relative to a group with no such constraint.

### Methods

One group was instructed to move straight to the target after it appeared, with no additional time constraints before moving (FREE, N = 19). The other group (WAIT_PLAN1, N = 17) was instructed to wait until they heard a high-pitched tone (1,000 Hz, 500-ms duration) that served as a go-signal. Based on previous work indicating that participants were able to aim 90° away from a visual target within ~1.3 s (McDougle & Taylor, 2019), we chose a 2.5-s wait interval to provide ample planning time for the 45° rotation task at hand. The go-signal was presented after this wait interval.

### Results

Data from one participant of the FREE group were excluded due to a large number of irregular trials (21% of premature movement initiations, moving too fast or too slow). Including this participant in the analyses (not shown) did not alter the results qualitatively.

**Figure 2:**
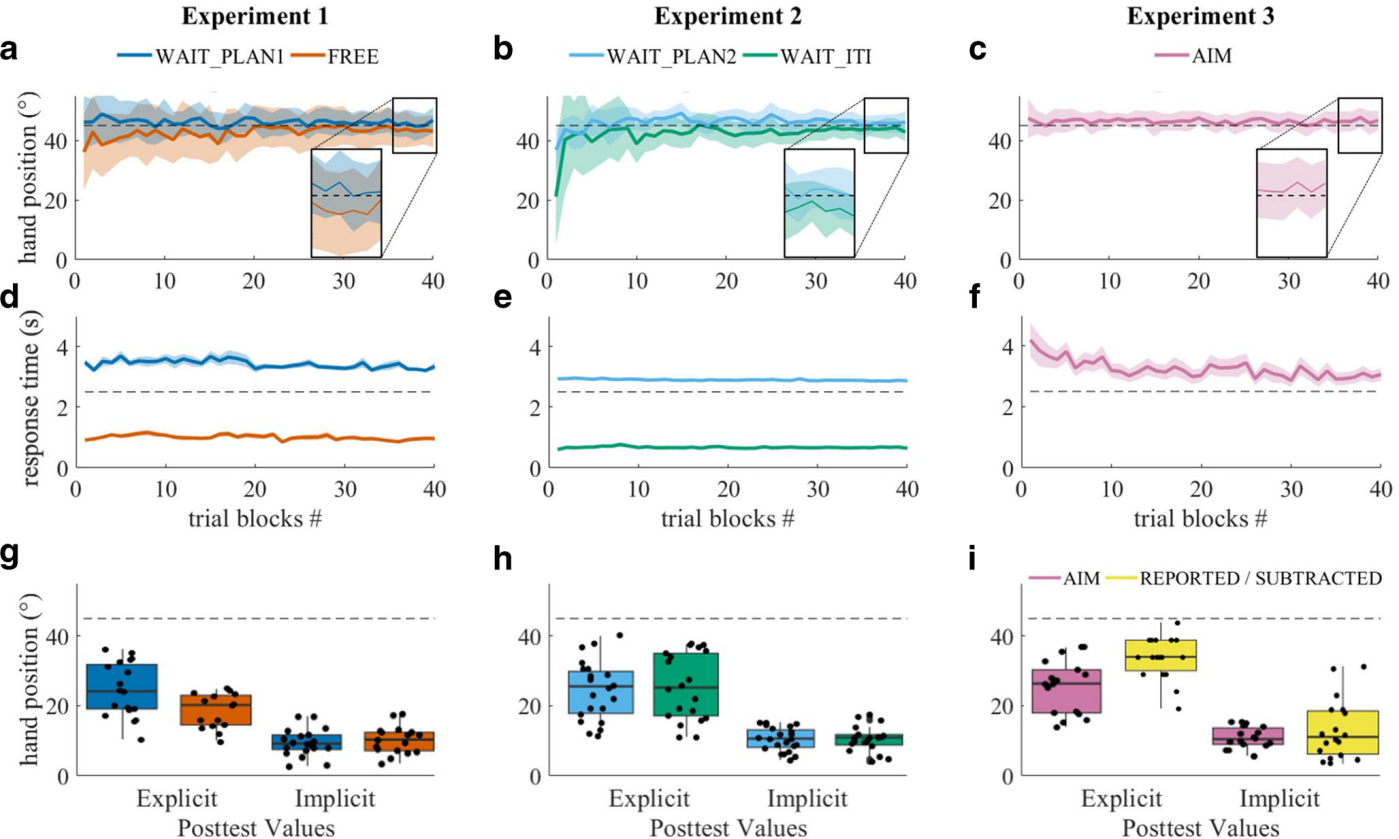
Mean hand direction (panels A-C) and mean movement response times (panels D-F) during practice plotted separately by experiments and groups. Panel G-I show the median hand direction during explicit and implicit posttests, separately and the individual data from single participants. The horizontal dashed lines in panels A-C and H-I indicate ideal compensation for the 45° cursor rotation. In panels D-F, they indicate the imposed waiting times of 2.5 seconds in the WAIT_PLAN groups. Shaded error bands in panels A-F represent standard deviation of the mean.

As shown in Fig. 2A, the FREE group displayed the typical incomplete asymptote (M = 41.15, SD = 8.28) (Table 1), whereas the WAIT_PLAN1 (M = 46.66, SD = 5.85) group achieved a greater asymptote (W = 244, p = 0.001, r = -0.42, CI = [-0.67, -0.13]). Hand directions late during practice were significantly less than 45° in the FREE (W = 32.5, p = 0.02 r = -0.61 CI = [-0.84, -0.21]) group, while the WAIT_PLAN1 group did not differ significantly from 45° (W = 108, p = 0.62, r = 0.12, CI = [-0.33, 0.53]) (Table 2).

**Table 1:** Mean and standard deviation for each experimental group at asymptote level, for the explicit judgment and the implicit after-effect post-tests

Group	Mean	SD
Asymptote		
FREE	41.15	8.28
WAIT_PLAN1	46.66	5.85
WAIT_PLAN2	46.33	3.99
WAIT_ITI	43.96	3.01
AIM	46.63	4.12
Explicit Judgment		
FREE	24.78	5.45
WAIT_PLAN1	30.65	8.33
WAIT_PLAN2	30.88	10.21
WAIT_ITI	30.53	8.57
AIM	28.32	10.95
Implicit After-Effects		
FREE	9.99	3.81
WAIT_PLAN1	9.35	3.67
WAIT_PLAN2	7.63	3.87
WAIT_ITI	8.45	4.77
AIM	8.87	3.29

**Table 2:** Parameters for Wilcoxon’s rank-sum tests between groups (compared groups are separated with a comma) and against 45° (complete compensation). Two-sided alternatives are represented with an unequal sign (≠ ), directed hypotheses are marked with a greater or smaller than symbol (> or <).

Wilcoxon’s rank-sum Test	W	p	Effect size r	95% confidence interval
Asymptote				
FREE, WAIT_PLAN 1	244	0.001	-0.42	[-0.67, -0.13]
Free < 45	32.5	0.02	-0.61	[-0.84, -0.21]
WAIT_PLAN1 ≠ 45	108	0.62	0.12	[-0.33, 0.53]
WAIT_PLAN2, WAIT_ITI	311	0.01	-0.34	[-0.59, -0.05]
WAIT_PLAN2 ≠ 45	235	0.28	0.25	[-0.18, 0.66]
WAIT_ITI < 45	63	0.02	-0.44	[-0.75, -0.05]
AIM > 45	125	0.05	0.41	[-0.08, 0.75]
Explicit Judgment				
FREE, WAIT_PLAN 1	83	0.04	-0.36	[-0.62, -0.031]
WAIT_PLAN2 WAIT_ITI	231	0.79	0.04	[-0.25, 0.36]
FREE, WAIT_PLAN2	85	0.03	-0.37	[-0.63, -0.06]
FREE, WAIT_ITI	93	0.03	-0.37	[-0.63, -0.08]
AIM, FREE	197.5	0.03	0.39	[0.05, 0.60]
AIM, WAIT_PLAN1	160.5	0.76	-0.05	[-0.36, 0.27]
AIM, WAIT_PLAN2	160	0.57	-0.09	[-0.39, 0.22]
AIM, WAIT_ITI	190.5	0.85	-0.03	[-0.34, 0.28]
AIM ≠ 45	0	< 0.01	-0.88	[-0.88, -0.87]
Implicit After-Effects				
FREE, WAIT_PLAN 1	179	0.59	0.09	[-0.24, 0.39]
WAIT_PLAN2, WAIT_ITI	214	0.89	-0.02	[-0.34, 0.36]
FREE, WAIT_PLAN 2	227.5	0.08	0.29	[-0.02, 0.58]
FREE, WAIT_ITI	256.5	0.05	0.34	[0.03, 0.62]
AIM, FREE	140.5	0.69	-0.07	[-0.39, 0.27]
AIM, WAIT_PLAN1	167.5	0.93	-0.02	[-0.35, 0.31]
AIM, WAIT_PLAN2	221	0.24	0.19	[-0.11, 0.52]

In the explicit judgment test (Fig. 2G), the FREE group estimated the rotation to be significantly smaller relative to the WAIT_PLAN1 group (W = 83, p = 0.04, r = -0.36, CI = [-0.62, -0.031]). Implicit after-effects (Fig. 2G) did not differ significantly between the groups (W = 179, p = 0.59, r = 0.09, CI = [-0.24, 0.39]) (Table 2).

### Discussion

Forcing participants to prolong their response time before movement onset on each trial led to an increase in asymptotic learning. Furthermore, this also led to an increase in accumulated explicit knowledge. While these results are consistent with our speed-accuracy tradeoff hypothesis, they cannot rule out an unspecific effect of time on task.

## Experiment 2

To further investigate whether the elevated asymptote from Experiment 1 was a nonspecific effect of time or in fact due to longer planning times, Experiment 2 aimed to delineate this by comparing two groups with matched intertrial intervals. According to the speed-accuracy tradeoff hypothesis, we predicted that, similar to Experiment 1, the outcome measure of final hand position would show less residual error in a group with prolonged response time compared to a group with an imposed post-movement intertrial interval even though total trial length was matched.

### Methods

Experiment 2 manipulated the trial duration in two groups: the WAIT_PLAN2 group (N = 22) was a replication of the WAIT_PLAN1 group in Experiment 1. Participants in the second group (WAIT_ITI, N = 20) could initiate movements as soon as the target had appeared on the screen replicating the planning interval of the FREE group from Experiment 1. Critically, the WAIT_ITI experienced an additional 2.5 s waiting period after the presentation of the endpoint feedback. Thus, the two groups, WAIT_PLAN2 and WAIT_ITI, had matched trial lengths but different planning intervals. During the 2.5-s inter-trial delay in the WAIT_ITI group, only the target was visible on the screen and participants were told to maintain their final hand position.

### Results

Inserting waiting time into the planning phase led to an asymptote not significantly different from 45° (M = 46.33, SD = 3.99), whereas inserting the waiting time into the intertrial interval led to an asymptote significantly less than 45° (M = 43.96, SD = 3.01) (Table 1). Those two asymptotes were significantly different from each other (W = 311, p = 0.01, r = -0.34, CI = [-0.59, -0.05]) (Table 2).

On the post-test for explicit knowledge (Fig. 2H), the temporal locus of the additional waiting time did not have a significant effect: Both groups appeared to accumulate equivalent amounts of explicit knowledge (W = 231, p = 0.79, r = 0.04, CI = [-0.25, 0.36]), but showed greater explicit estimations than the FREE group in Experiment 1 (FREE, WAIT_PLAN2: W = 85, p = 0.03, r = -0.37, CI = [-0.63, -0.06]; FREE, WAIT_ITI: W = 93, p = 0.03, r = -0.37, CI = [-0.63, -0.08]), whose trial structure did not contain any additional waiting interval. Furthermore, after-effects in Experiment 2 did not differ significantly between groups (W = 214, p = 0.89, r = -0.02, CI = [-0.34, 0.36], Fig. 2H).

### Discussion

The absence of significant asymptotic error in the WAIT_PLAN2 group replicated the effect of additional planning time observed in Experiment 1. Comparing the WAIT_ITI group with the FREE group across experiments suggests that extending the intertrial interval may have had an unspecific effect on learning as indicated by greater explicit learning in the WAIT_ITI group.

Importantly, the significant difference between groups and the fact that the WAIT_ITI group displayed an incomplete asymptote shows that most of the benefit of added time in Experiment 1 was not a mere consequence of a prolonged intertrial interval, suggesting a specific benefit of additional time for movement planning in line with our speed-accuracy tradeoff hypothesis.

## Experiment 3

As both preceding experiments hinted toward an unspecific effect of time on task on learning due to accumulating more explicit knowledge, this experiment sought to account for the possibility that it is in fact not time *per se* but the increased participation of explicit processes that raises the level of asymptote. To this end, we used the reporting paradigm as this procedure requires active explicit engagement during the planning interval. We hypothesized that the dependent variable of final hand position would thus show close to no residual error.

### Methods

A single group of participants (AIM group, N = 18) was asked to report their aiming direction prior to movement initiation (Bond & Taylor, 2015; McDougle et al., 2015; Taylor et al., 2014). The participants in this group saw a numbered ring of visual landmarks. The numbers were arranged at 5.63° intervals with the current target positioned at the 0° position. Clockwise the numbers became larger and counterclockwise the numbers became smaller (up to 32°, -32°, respectively), forming a circle 20 cm in diameter. Participants were instructed to verbally report the number they were aiming their reach at before moving (see Taylor et al., 2014, for further information on this task). Verbal reports were manually registered by the experimenter on each reporting trial. In Experiment 3, baseline training included three additional blocks in which participants had to report their aiming direction prior to movement onset.

### Results

Participants in the AIM group completely compensated the rotation. Adaptive shifts in hand positions were significantly larger than 45° (M = 46.63, SD = 4.12, W = 125, p = 0.05, r = 0.41, CI = [-0.08, 0.75]) (Table 2), suggesting that adaptation at asymptote was complete and, in fact, some participants overcompensated for the rotation (Fig. 2C). Explicit judgments of required compensation (Fig. 2I) were significantly less than 45° (M = 28.32, SD = 10.95, W = 0, p < 0.01, r = -0.88, CI = [-0.88, -0.87]) (Table 2).

To test whether the reporting task influenced the outcome of the explicit judgment tests, we compared the post-test values between the AIM group and those of the other groups in Experiments 1 and 2. There was a significant difference in the explicit judgments between the AIM group and the FREE group from Experiment 1 (W = 197.5, p = 0.03, r = 0.39, CI = [0.05, 0.60]). Across the AIM group and WAIT_PLAN2 (W = 160.5, p = 0.76, r = -0.05, CI = [-0.36, 0.27]) and WAIT_ITI (W = 190.5, p = 0.85, r = -0.03, CI = [-0.34, 0.28]) groups in Experiment 2, there were no differences in the explicit judgment tests (Table 2).

### Discussion

By instructing participants to verbally report their movement aim prior to movement execution trial-by-trial (Taylor et al., 2014), we potentially primed the explicit component of adaptation by getting subjects to attend to angular deviations. We reasoned that this would serve as an opportunity to replicate our findings, in that requiring active explicit reporting also extends the planning interval. Our results suggest that experimentally querying the explicit process of adaptation does not qualitatively alter the explicit learning balance, but does act to improve the adaptation asymptote by promoting planning and prolonging the movement planning interval.

## Concluding discussion

This study was designed to investigate whether previously reported findings of incomplete asymptotic visuomotor learning may be reframed as an instantiation of the speed-accuracy tradeoff. In line with this hypothesis, artificially prolonging the waiting period prior to goal-directed movement onset elevated asymptotic learning and appeared to eliminate residual errors. This benefit was specific to prolonging motor planning (prior to a go-signal). Prolonging the interval between visual feedback and the start of the next trial did not provide the same benefit. Our results suggest that time-consuming planning processes are a major cause of incomplete asymptotic learning.

Why did hasty planning result in consistent undershooting rather than, for example, increased movement variability? We suggest that parametric mental computations might explain this phenomenon: In visuomotor rotation tasks, participants’ response times increase linearly with the magnitude of imposed rotations (Georgopoulos & Massey, 1987; McDougle & Taylor, 2019), reflecting a putative mental rotation process (Shepard & Metzler, 1971). A previous study by McDougle and Taylor (2019) demonstrated that reaction time in a free condition appeared to decompose into a ~1-s baseline reaction time plus ~200 ms for a ~45° mental rotation (their Fig. 4b). Thus, the potential savings by rotating incompletely may seem small; however, it is consistent with our response-time results (Fig. 2D), and it is also consistent with decision-making research that shows similar amounts of time being saved in reward-based speed-accuracy tradeoff tasks (Thura, Cos, Trung, & Cisek, 2014). Interestingly, in another experiment by McDougle and Taylor (2019), participants reliably rotated movements to around ~75° when a forced total reaction time of ~350 ms was imposed for a 90° perturbation. This may indicate that urgency imposed by the forced response task independently modulates the baseline preparation time. Overall, this mental rotation interpretation is further supported by the results of our third experiment, in which emphasizing the application of explicit aiming strategies prior to movement initiation led to qualitatively similar asymptotic learning as in the groups with prolonged response times. Finally, we note that delaying movement initiation did not only cause full compensation, but induced overcompensation, suggesting perhaps that implicit processes superimposed onto an accurate explicit rotation strategy may have caused reach angles to drift, gradually adapting the hand further in the direction of compensation (cf. Mazzoni & Krakauer, 2006).

The idea of a speed-accuracy tradeoff prematurely interrupting putative mental rotation processes during motor planning also provides an explanation for previously observed age-related differences in visuomotor learning: Hegele and Heuer (2013) used explicit instructions and cognitive pretraining prior to learning a novel visuomotor rotation to boost explicit knowledge of the transformation. Older adults with full explicit knowledge of the transformation turned out to be less efficient in applying it for strategic corrections of their aiming movements. This age-related difference with respect to the behavioral exploitation of explicit knowledge became manifest only when participants had almost perfect explicit knowledge, but not when they had only poor explicit knowledge and showed minimal strategic adjustments. Given the present results, one could speculate that the reduced exploitation of explicit knowledge for strategic corrections in older participants is due to a combination of age-related slowing in mental rotation and the premature termination of (slowed) mental rotation during motor planning.

Traditionally, the incomplete asymptote phenomenon has been explained by state-space models of adaptation (Smith et al., 2006). As subsequent studies indicated that this model alone is insufficient for explaining incomplete asymptotic behavior, alternatives were proposed: among others, that spatial error-based learning processes suppress other mechanisms that could drive full compensation (Shmuelof et al., 2012; Vaswani et al., 2015). In our study, participants in all groups received similar spatial error feedback. Thus, a potential suppression should have affected all groups equally, making this explanation insufficient to explain the modulations in asymptote we observed.

Another approach to the state-space model suggests that residual errors in adaptation paradigms are caused by implicit processes that tune the motor system’s sensitivity to errors until it reaches an equilibrium with constant forgetting (Albert et al., 2019). These authors manipulated the variability of the perturbation and found that residual errors increase with the perturbations’ variance. Without having considered this a priori, we note that our hypothesis could potentially be adapted to account for these variations in asymptote (e.g., experiencing perturbation variability could affect the benefit that learners expect from planning, and thus the time they spend on it). However, in one experiment that study also showed a speed-accuracy tradeoff by obtaining larger residual errors when the reaction time was artificially shortened compared to free response times, regardless of the variance of perturbation. Thus, we believe that additional planning time is an essential element in eliminating residual errors to achieve full compensation, though it is likely not the only thing determining the exact asymptotic value.

Moreover, we also note that consistent undershooting relative to the perturbation, as observed here and in previous studies, is critically not seen in experimental paradigms designed to isolate the implicit component of visuomotor adaptation (Morehead et al., 2017) – indeed, even when rotational perturbations are as small as ~1.75°, implicit adaptation appears to asymptote around ~15° (Kim et al., 2018). In the current study, results from the implicit post-test were unaffected by changes in the response-time interval. Thus, it may be that incomplete compensation relative to the visual error mainly involves explicit cognitive processes that succumb to speed-accuracy tradeoffs, whereas asymptotic dynamics of the implicit system require a separate explanation.

Recent accounts have framed motor planning as a time-consuming optimization process from which a reduction in movement accuracy arises naturally when constraints are imposed (Al Borno et al., 2019). Our findings suggest that similar principles apply when one is intentionally choosing to perform a movement in another direction than the one implied by the target presented, and that learners naturally constrain their planning time even in seemingly unconstrained conditions. Haith and colleagues (Haith et al., 2016) recently showed that movement preparation and initiation are independent, i.e., that, instead of complete preparation triggering movement initiation, humans appear to determine a time for movement initiation based on when they expect planning to be completed. This view naturally implies the possibility for premature movement initiation. The planning time chosen may therefore trade off the achieved accuracy within a given time and the urgency to move on (Churchland et al., 2008; Cisek et al., 2009; Thura & Cisek, 2017).

Many of the common explanations for incomplete asymptote outlined above imply that it is a fundamental property of learning. Psychology and kinesiology traditionally distinguish performance effects (the behavioral act of executing a skill at a specific time in a specific situation) from learning effects (the change in the unobservable underlying capability to perform a skill, which is indirectly inferred from a relatively permanent improvement of performance). For example, with respect to the asymptotic reaching behavior of two groups in our experiments, their underlying knowledge could be identical while retrieval processes in specific test conditions can lead to different performance profiles (Magill & Anderson, 2017; Schmidt & Lee, 2011). Even though our experiments were not specifically designed to distinguish learning from performance, our findings suggest that both may contribute to an incomplete asymptote in adaptation: If our results reflected a performance effect alone, the manipulation should have affected behavior in the adaptation phase but not in the post-test results. In Experiment 1, however, explicit estimates of the rotation magnitude were increased with added response time, suggesting that perhaps some of the benefit of longer response times may be due to learners honing their explicit knowledge. However, the observation that explicit knowledge was similarly increased regardless of whether additional time was added at the beginning or end of a trial in Experiment 2 indicates that this learning effect may be a non-specific consequence of longer intertrial intervals (it is), and that the remaining increase in asymptote is indeed a performance effect. A recent paper analyzing preparatory neural states in rhesus monkeys performing visuomotor learning tasks also found that longer preparation times not only yielded smaller variance on the current trial, but also smaller errors on the subsequent trial, supporting a learning effect (Vyas et al., 2020). Future research could attempt to better delineate learning from performance effects in human motor adaptation. Moreover, the post-tests reported here should be interpreted with caution: Recent work suggests that measurements of explicit visuomotor learning components are contingent on the methodology used (Maresch, Werner, & Donchin, 2020).

Lastly, we emphasize that we are not claiming that other learning mechanisms cannot contribute to asymptotic behavior (Albert et al., 2019; Emken et al., 2007), nor that a state-space model with gradual decay towards zero is invalid (Brennan & Smith, 2015). What we do suggest is that a potentially major aspect determining the magnitude of asymptotic errors in visuomotor learning is a speed-accuracy tradeoff. Since this decision process is likely to be relevant across a broader range of motor tasks, we speculate that our results extend beyond motor adaptation, and that simple interventions, like explicitly prolonging response times to allow for complete planning, could improve asymptotic performance in a range of motor learning tasks.
